# Epigenetic Input Dictates the Threshold of Targeting of the Integrin-Dependent Pathway in Non-small Cell Lung Cancer

**DOI:** 10.3389/fcell.2020.00652

**Published:** 2020-07-22

**Authors:** Yang Zhang, Kai Cheng, Bingwei Xu, Junfeng Shi, Jun Qiang, Shujin Shi, Yuanqin Yi, Hongxia Li, Tengchuan Jin, Ruihua Guo, Yadi Wu, Zeyi Liu, Xiaowei Wei, Jian-An Huang, Xiuwei H. Yang

**Affiliations:** ^1^Department of Respiratory Medicine, First Affiliated Hospital of Soochow University, Suzhou, China; ^2^Department of Pharmacology and Nutritional Sciences, University of Kentucky, Lexington, KY, United States; ^3^Markey Cancer Center, University of Kentucky, Lexington, KY, United States; ^4^Department of Pathology, Nanjing Jinling Hospital, Nanjing University School of Medicine, Nanjing, China; ^5^Department of Oncology, Nanjing Medical University, Nanjing, China; ^6^Ministry of Agriculture, Freshwater Fisheries Research Center, Chinese Academy of Fishery Sciences, Wuxi, China; ^7^Fisheries College, Nanjing Agricultural University, Wuxi, China; ^8^Cancer Institute, The First Affiliated Hospital of China Medical University, Shenyang, China; ^9^Laboratory of Structural Immunology, Division of Life Sciences and Medicine, University of Science and Technology of China, Hefei, China; ^10^College of Food Science and Technology, Shanghai Ocean University, Shanghai, China

**Keywords:** lung cancer, FAK, BRD4, integrins, KRAS targeted therapy

## Abstract

We investigated the therapeutic potential of targeting integrin/FAK-dependent signaling, an adhesion receptor-mediated pathway that has been increasingly linked to non-small cell lung cancer (NSCLC) malignancy. Our analysis of the TCGA cohort showed that a subset of pro-tumorigenic integrins, including α1β1, α2β1, α3β1, α5β1, and α6β4, were frequently amplified or upregulated at the genomic or mRNA level in KRAS or EGFR mutation/overexpression-enriched adenocarcinomas. These alterations appeared complementary, correlated with poor patient survival (*p* < 0.0072), and were collaborative with KRAS mutation-coupled αv integrins (*p* < 0.00159). Since integrin/FAK-dependent signaling is tightly coupled with normal human physiology, we sought to use a synthetic lethal-type targeting comprising of VS-6063, a chemical inhibitor of integrin-mediated FAK activity, and A549 cells, which carry a KRAS mutation and EGFR overexpression. Our screening analysis revealed that JQ1 and IBET-762, inhibitors of epigenetic reader BRD4, and LBH589, a pan inhibitor of histone deacetylases (HDACs), exhibited synergy with VS-6063 in mitigating tumor cell viability. This epigenetic link was corroborated by strong effects of additional inhibitors and RNAi-mediated knockdown of FAK and BRD4 or its downstream effector, c-Myc. Low doses of JQ1 (≤0.5 μM) markedly escalated efficacy of VS-6063 across a panel of 10 NSCLC cell lines. This catalyst-like effect is in line with the oncogenic landscape in the TCGA cohort since c-Myc falls downstream of the KRAS and EGFR oncogenes. Mechanistically, co-inhibiting the integrin-FAK and BRD4/c-Myc axes synergistically induced apoptotic cell death and DNA damage response, and impaired stemness-associated tumorsphere formation. These effects were accompanied by a marked inhibition of Akt- and p130Cas/Src-dependent signaling, but not Erk1/2 activity. Meanwhile, JQ1 alone or in combination with VS-6063 attenuated cell-cell adhesion and extracellular matrix (ECM)-dependent cell spreading, which is reminiscent of phenotype induced by malfunctional E-cadherin or integrins. Paradoxically, this phenotypic impact coincided with downregulation of epithelial-mesenchymal transition (EMT)-inducting transcription factor ZEB1 or Snail. Finally, we showed that the effect of the VS-6063/JQ1 combination was nearly equivalent to that of VS-6063 plus Carboplatin or Osimertinib. Overall, our study indicates that the integrin/FAK and BRD4/c-Myc axes cooperatively drive NSCLC virulence, and a co-targeting may provide a line of therapy capable of overcoming EGFR/KRAS-driven malignancy.

## Introduction

Lung cancer, particularly non-small cell lung cancer (NSCLC), is one of leading causes of cancer-related deaths worldwide, despite recent progress in targeted therapies and early diagnosis for the disease (Kleczko and Heasley, 2018; Cao and Chen, 2019; Hinz et al., 2019; Li W.Y. et al., 2019). Extensive genome-wide studies have revealed that the prognosis of NSCLC patients is particularly related to point mutations or gene copy alterations of two groups of oncogenic drivers: receptor tyrosine kinase (RTK) and RAS (Cancer Genome Atlas Research Network, 2012; Chen et al., 2017). Tumors exhibiting oncogenic activation of RTKs, particularly EGFR, are highly susceptible to inhibition by small molecule inhibitors in the clinic, notably Gefitinib and Erlotinib (Hirsch et al., 2016; Vestergaard et al., 2018). More than 30% of NSCLC patients, however, carry active KRAS mutations and are confronted with limited therapeutic options with a poor clinical outlook (Brugger et al., 2011; Vestergaard et al., 2018). The G12/C codon change appears particularly enriched in these KRAS mutant NSCLC adenocarcinomas and promotes epithelial-mesenchymal transition (EMT)-linked disease progression (Patricelli et al., 2016; Janes et al., 2018). While the chemical inhibitor-based targeting of this mutational KRAS is under clinical trial investigation (Yuan et al., 2018), there remains a lack of an effective therapeutic strategy for treating NSCLC tumors carrying non G/C substitutions of KRAS or with co-activation of other oncogenes or tumor suppressors (Yuan et al., 2018; Skoulidis and Heymach, 2019). Hence, more targeted therapies are still urgently needed to enhance NSCLC treatment.

Integrins, a large family of heterodimeric cell adhesion receptors, are increasingly implicated as strong mediators of NSCLC malignancy (Cabodi et al., 2010). Upon engagement with their extracellular matrix (ECM) ligands, integrins mediate lung tumorigenesis and metastasis in both KRAS-dependent and independent manners (Yoshimasu et al., 2004; Singh et al., 2009; Caccavari et al., 2010; Lawson et al., 2010; Morello et al., 2011; Konstantinidou et al., 2013). Notably, the collagen-binding α1β1 and α2β1 integrins, vitronectin/Thrombospondin-binding αvβ6, αvβ6, and αvβ8 integrins, as well as laminin-binding α3β1 and α6β4 integrins, all appear engaged in the regulation of tumor cell proliferation and survival, and the epithelial mesenchymal transition (EMT) program (Prudkin et al., 2009; Singh et al., 2009; Caccavari et al., 2010; Stipp, 2010). These pro-tumorigenic functions seem linked to the activation of intracellular focal adhesion kinase (FAK) and its downstream signaling through Akt-, Src- and small GTPase (e.g., Rac1, RhoA)-dependent pathways (Thanapprapasr et al., 2015). Additionally, the integrin/FAK-dependent signaling appears to act in synergy with RTKs (e.g., EGFR, ErbB2, and c-Met) to impact NSCLC malignancy and susceptibility to current therapies (Yoshimasu et al., 2004; Caccavari et al., 2010; Lawson et al., 2010). Together, this evidence implies that the integrin-FAK-dependent pathway plays a crucial role in tumor onset and progression of NSCLC, and offers a line of promising therapeutic targets.

To date, the targeting of integrin-dependent functions and signaling in human cancer has been largely implemented via function-blocking monoclonal antibodies or small chemical inhibitors against active FAK, particularly its Y^397^ autophosphorylation^15,20,21^. Given that integrins are frequently co-upregulated and functionally redundant in tumor cells, targeting of any single integrins is likely insufficient to eliminate their impact on cancer malignancy. In contrast, nearly all integrins signal through activation of FAK and its associated downstream pathways, leading to accelerated tumor development and progression. As a result, diverse chemical inhibitors of FAK have been developed and tested for therapeutic potential in the context of disrupting integrin-dependent signaling through PI3K/Akt-, Src/p130-, and RhoA/Rac1-mediated pathways, associated impact on tumor cell functions and behaviors, and tumorigenesis and metastasis (Cordes and Park, 2007; White and Muller, 2007; Caccavari et al., 2010). Recently, some FAK inhibitors have been investigated for their efficacy in mitigating NSCLC response to radiation, chemotherapies, and targeted therapies such as RTK inhibitors or PDL/PDL1-based immunotherapies (Jiang et al., 2016). While this evidence supports the therapeutic potential of FAK inhibitors, these agents often exhibit limited efficacy during clinical trials, including those with NSCLC patients (Shapiro et al., 2014). Thus, new studies are needed to develop an effective integrin-FAK-based therapy against human NSCLC.

The current study aims to pursue synthetic lethal-type targeting of this pathway for NSCLC, particularly for the subset of adenocarcinomas exhibiting KRAS mutations. We performed chemical inhibitor screening to define candidate allies for the integrin/FAK-mediated signaling pathway in NSCLC tumor cells. We subsequently interrogated specificities of these candidates at the molecular and cellular levels through assessment of their co-expression with FAK in NSCLC patient cohorts. Results from our studies demonstrate that the BRD4-centered epigenetic network modulates the pro-tumorigenic role of the integrin/FAK pathway in both c-Myc-dependent and c-Myc-independent manners in NSCLC. A co-inhibition of these two axes cooperatively suppressed tumor cell survival, induced DNA damage response, and disrupted their capacity to develop the stemness-associated tumorspheres. As such, our data supports the notion that co-targeting the integrin-dependent pathway and BRD4-associated epigenetic network is an alternative therapy against NSCLC malignancy, particularly for patients carrying KRAS mutations.

## Materials and Methods

### Cell Culture, Antibodies and Chemical Inhibitors

A panel of authenticated human lung carcinoma cell lines used in the study, including A549, H460, H520, H1299, SK-MES-1, H2030, H2122, H1975, HCC827, and PC9, along with 16BHE, an immortalized human bronchial epithelial cell line, were obtained from ATCC (Manassas, VA, United States). All cell lines were cultured in RPMI 1604 or DMEM (Invitrogen) supplemented with 5–10% FBS (Sigma-Aldrich, St; Louis, MO, United States) under 37°C and 5% CO_2_. Mammary epithelium medium was purchased from Lonza (Basel, Switzerland). During the study all cell lines were periodically examined for Mycoplasma contamination by PCR analysis (Cheung et al., 2011).

The sources of antibodies and chemical inhibitors are described in a prior study (Xu et al., 2017). Additional antibodies, including BRD4 and phosphorylated or γH2AX, were purchased from Cell Signaling Technology (Danvers, MA, United States). Osimertinib was obtained from Selleckchem (Houston, TX, United States).

### RNAi Oligos and Transfection

Gene knockdown was performed with siRNA oligos purchased from Cell Signaling Technology or Dharmacon (Boulder, Denver). The sequences of RNAi oligos were provided in a recent study (Xu et al., 2017). The transient silencing of gene or protein expression was conducted by use siRNA oligos and Lipofectamine 2000 (Thermo Fisher, Waltham, MA, United States). The shRNA-mediated stable knockdown was carried out using lentiviral infection and subsequent selection with a combination of Puromycin and GFP-based cell sorting on flow cytometry (Xu et al., 2017).

### Functional Assays

Tumor cell viability was measured using MTT assay (Xu et al., 2017) and percentage of viable cells was calculated relative to 0.1% DMSO control. Changes in cell cycle were assessed by flow cytometry-based analysis of Propidium iodide staining of tumor cells after being treated with various doses of chemical inhibitors for 36 h in the presence of 10% FBS. The portion of apoptotic cell death was assessed by analyzing proportion of treated with indicated inhibitors for 48–72 h in the presence of 5–10% FBS, stained with a combination of propidium iodide and APC-conjugated Annexin V (10 μg/ml, BioLegend, San Diego, CA, United States), and analyzed by flow cytometry at the core facility.

### Analysis of Cell Signaling and Immunoblotting

Changes in cell signaling pathways were examined by treating the 70–80% confluent cell culture with vehicle (largely 0.1–0.4% DMSO) and diverse chemical inhibitors or RNA oligos for 17–48 h. After washing in PBS or plain medium, treated cells were lysed in RIPA buffer supplementary protease inhibitor cocktail (Sigma-Aldrich, St. Louis, MO, United States), PMSF and Na_3_VO_4_ (Xu et al., 2017). Immunoblotting was carried out by incubating lysates with primary and secondary antibodies, followed by detection with the Chemoluminescence kit (Thermo-Fisher). β-actin was assessed as a loading control. In parallel, cell signaling was analyzed with human phosphor-kinase antibody arrays according to manufacturer instructions (R&D Biosystems, Minneapolis, MN, United States).

### Tumorsphere Formation Assay

The effect on tumorsphere formation was assayed as described in our prior study (Li H. et al., 2019). In brief, 1.0 × 10^3^ tumor cells were seeded into a single well of 24-well ultra-low adhesion plate overnight in stem cell medium, followed by treatment with chemical inhibitors for 5 days before being imaged microscopically. Tumor cells were seeded into ultralow adhesion 24-well plates and treated with inhibitors for 5 days prior to imaging on a NIKON Eclipse microscope work station.

### IHC and Bioinformatic Mining of TCGA Patient Cohort

Our IHC analysis was performed on paraffin-embedded lung tumors collected from a patient cohort recently diagnosed or treated at the Jinling Hospital (Nanjing). The scoring of antibody staining was carried out as descried in our prior studies (Yang et al., 2008; Zhou et al., 2015). In brief, the values for protein expression in tumor tissues were obtained through calculation of percentage of positively stained tumor cells x score of staining intensity. Gene amplification and mRNA expression in the breast cancer patient cohort at the TCGA database was analyzed through the c-Bio Portal platform (Barretina et al., 2012).

### Statistical Analyses

χ^2^ analysis was adopted to evaluate degree of co-expression of FAK and BRD4 or c-Myc in patient tumor biopsies. A two-sample *t*-test was used to assess differences in tumor size or weight between groups. Kaplan-Meier curves and Log-rank tests were employed to compare survival time between groups.

## Results

### Association Between Integrin Deregulation and Clinical Outcomes in NSCLC

**FIGURE 1:**
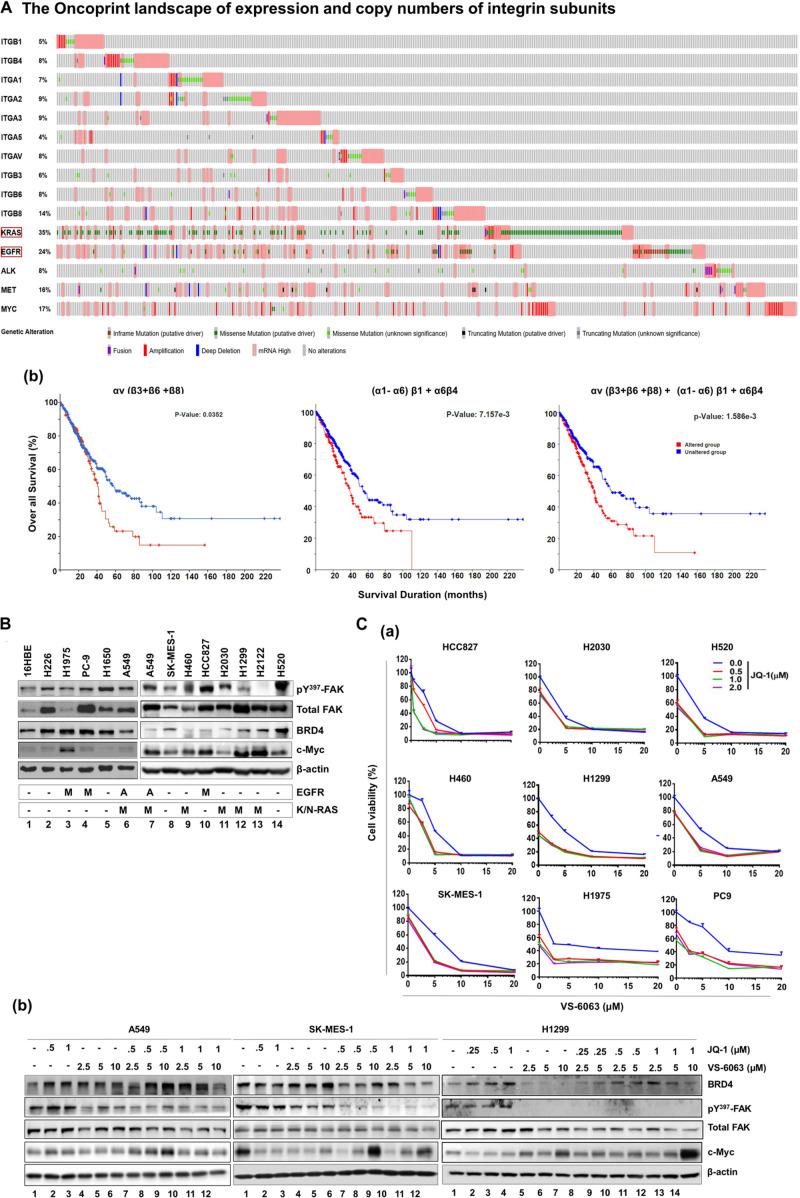
Identification of the link between the integrin-FAK axis and BRD4 in NSCLC. **(Aa)** The oncoprint map of genomic and mRNA deregulation of major epithelial cell/mesenchymal cell-associated integrins and a list of major oncogenes in adenocarcinomas of the NSCLC cohort (TCGA pan alters, Nature 2014, *n* = 503). **(b)** Association between deregulation of integrin subsets and patient survival. *P* values from Log-rank tests are shown. The *altered group* includes number of patient biopsies exhibiting genomic or mRNA changes (relative to the diploid group) for indicated subset of integrin genes, ranging from missense and truncating gene mutations, and gene fusion, amplification or deletion to altered mRNA level. Profiling response to the co-inhibition of FAK and BRD4 across NSCLC cell lines. **(B)** Expression profile of active and total FAK, BRD4 and c-Myc across a panel of representative NSCLC cell lines. Tumor cells were lysed in RIPA buffer and immunoblotted with indicated antibodies. A: gene amplification; M: oncogenic mutation. **(C)** Effect of varying doses of FAK and BRD4 inhibitors viability for nine NSCLC cell lines and the immortalized 16HBE line (data not shown) **(a)** or treated for 24 h, lysed in RIPA buffer, and blotted with indicated antibodies **(b)**. Cell viability was determined by treating tumor cells with 0.1% DMSO or varying doses of VS-6063 and/or JQ1 for 72 h, followed by MTT analysis. Values: Mean ± SEM (*n* = 3) calculated as percentage of viable cells relative to 0.1% DMSO control. **(B,C)** β-actin was blotted for equal protein loading control.

To identify clinically relevant key members of the integrin family to NSCLC, we performed bioinformatic analysis on genomic and mRNA expression of solid-tumor-linked integrins in the NSCLC adenocarcinoma cohort in the TCGA database (Supplementary Figure S1A). Our analysis showed that nearly all integrins exhibited genetic mutation and alteration in gene copy number or mRNA level (Figure 1Aa). Next, we narrowed our analysis to a subset of pro-tumorigenic integrins based on recent studies: α1β1, α21, α3β1, α5β1, α6β4, αvβ3, αvβ6, and αvβ8 (Caccavari et al., 2010; Sulzmaier et al., 2014; Aboubakar Nana et al., 2019). These integrins exhibited frequent genomic alterations or elevated mRNA expression in 5–14% of adenocarcinomas in the cohort. These changes, to a large degree, appeared mutually exclusive. Given the close association between αv integrins and KRAS-mediated growth of lung adenocarcinomas (Prudkin et al., 2009; Singh et al., 2009), we examined the clinical outcomes of patients from this group (Figure 1A,b). These patients exhibited significantly poorer survival compared to their counterparts (*p* = 0.0352). A similar trend was detected for the population exhibiting alteration in α1β1, α21, α3β1, α5β1, and α6β4 (*p* = 0.00715) and the patient population exhibiting alteration in both sets of integrins (*p* = 0.0016). The lung adenocarcinomas exhibiting integrin upregulation were highly enriched in mutation and mRNA upregulation of KRAS or EGFR, and were linked to poor clinical prognosis compared to those carrying alterations in single oncogene (Supplementary Figure S1B). Together, these clinical analyses imply that integrins play a role in the NSCLC malignancy driven by oncogenic activation of KRAS and EGFR.

### The Link Between the Integrin-FAK Pathway and Epigenetic Network

Based on the clinical importance of integrins in NSCLC cells (Figure 1A), we examined their signaling roles through their key downstream effector, focal adhesion kinase (FAK) in A549 cells, which carried EGFR amplification and the KRAS mutation (G12 → S). Our initial analysis showed that tumor cell viability decreased by approximately 40%, when treated with ≤5.0 μM, VS-6063, a chemical inhibitor of active FAK which blocks its Y^397^ autophosphorylation (Supplementary Figure S2A). Given the limitation of clinical efficacy of this FAK inhibitor against epithelia-origin human cancers (Jones et al., 2015; Xu et al., 2017; Gerber et al., 2020), we examined the combinatorial inhibition strategy for this inhibitor-based targeting by performing cell viability-based chemical inhibitor screening. As shown in Supplementary Figure S2, two out of more than 30 anti-tumor chemical agents screened, the pharmacological agents of epigenetic drivers BRD4 (JQ1) and histone deacetylase (HDAC, LBH589) (Filippakopoulos et al., 2010), and the pro-survival Akt (MK-2206) and Bcl-2 (ABT-737), exhibited strong cooperative effects with VS-6063 in terms of cell viability inhibition. To our knowledge, our observation of the close link between the integrin-FAK axis and epigenetic network appears to be the first report for NSCLC cells, particularly those with amplification or oncogenic mutation of KRAS and EGFR. This finding was corroborated by the cooperative effect of other FAK and BRD4 inhibitors, BET-752 and VS-4718, in A549 cells (Supplementary Figure S3). There appeared to be a lack of apparent association between FAK and BRD4 or their downstream effector c-Myc across a panel of NSLC cell lines (Figure 1B). In addition, our observed link between the integrin-dependent signaling and Akt or Bcl2-mediated cell survival pathway was consistent with prior studies on FAK in multiple cancer types, particularly those exhibiting frequent FAK amplification (Sulzmaier et al., 2014; Xu et al., 2017).

**FIGURE 2:**
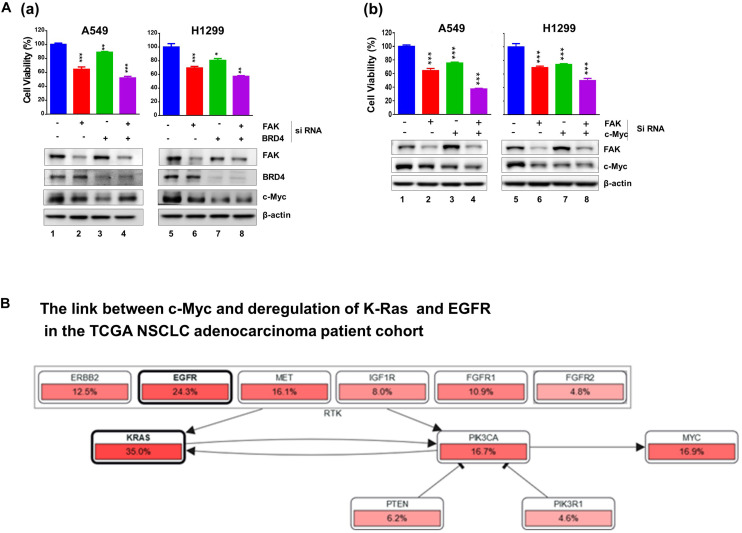
Effect of co-downregulation of FAK and BRD4 to c-Myc in NSCLC cells. **(A)** A549 and H1299 cells were subjected to RNAi oligo treatment for 24 h, replaced with fresh culture medium for 48 h, followed by analysis of cell viability using MTT assay (Mean ± SEM, *n* = 3). Extent of protein downregulation of FAK, BRD4 and c-Myc was assessed by immunoblotting. *p < 0.05; **p < 0.01; ***p < 0.005. **(B)** Upstream regulators of c-Myc in the TCGA NSCLC patient cohort.

Next, we profiled the combined effect for JQ1 and VS-6063 across a panel of representative NSCLC cell lines, (Figure 1C). Relative to each agent alone, the inhibitor combination displayed strong synergistic effect, particularly in the dose range of 0 < JQ1 < 1.0 μM and 0 < VS-6063 < 5-10 μM), in A549, HCC827, H520, H1299, SK-MES-1, and PC9, regardless of oncogenic status of EGFR or KRAS. This catalyst-like impact of JQ1 on tumor cell sensitivity to VS-6063 was in line with our recent study of another epithelia-originating cancer (Xu et al., 2017), even though the co-amplification of FAK and c-Myc rarely occurs in human NSCLC adenocarcinomas. Furthermore, the effects of these inhibitors were on target, as our immunoblotting analysis detected a marked decrease in active FAK in multiple cell lines (Figure 1C). Intriguingly, the effect of co-inhibition of FAK and BRD4 on c-Myc level appeared to vary with cell lines. c-Myc exhibited a marked decrease in SK-MES-1-line o, but not A549 and H1299, upon treatment with varying doses of inhibitors alone or in combination except a high dose of VS-6063 (10 μM) (Figure 1C). Meanwhile, there appears a decrease in total level of FAK under escalating doses of VS-6063, which presumably originates from concomitant proteasome-associated protein degradation (Sulzmaier et al., 2014). Moreover, this cooperative effect was partially recapitulated by analysis of simultaneously downregulating FAK and BRD4 or c-Myc (Figure 2A). These data are also consistent with the output from the pathway analysis of the TCGA dataset, where c-Myc sits downstream of KRAS and EGFR or other RTKs in the NSCLC cohort (Figure 2B).

Combined, our chemical inhibitor screening, gene knockdown and bioinformatic analyses consistently indicate a collaborative role of the integrin-FAK pathway and BRD4/Myc-linked epigenetic network in controlling tumor cell variability in NSCLC, regardless state of KRAS or EGFR.

### Functional Impact of Co-inhibition of the Integrin-FAK Axis and BRD4

**FIGURE 3:**
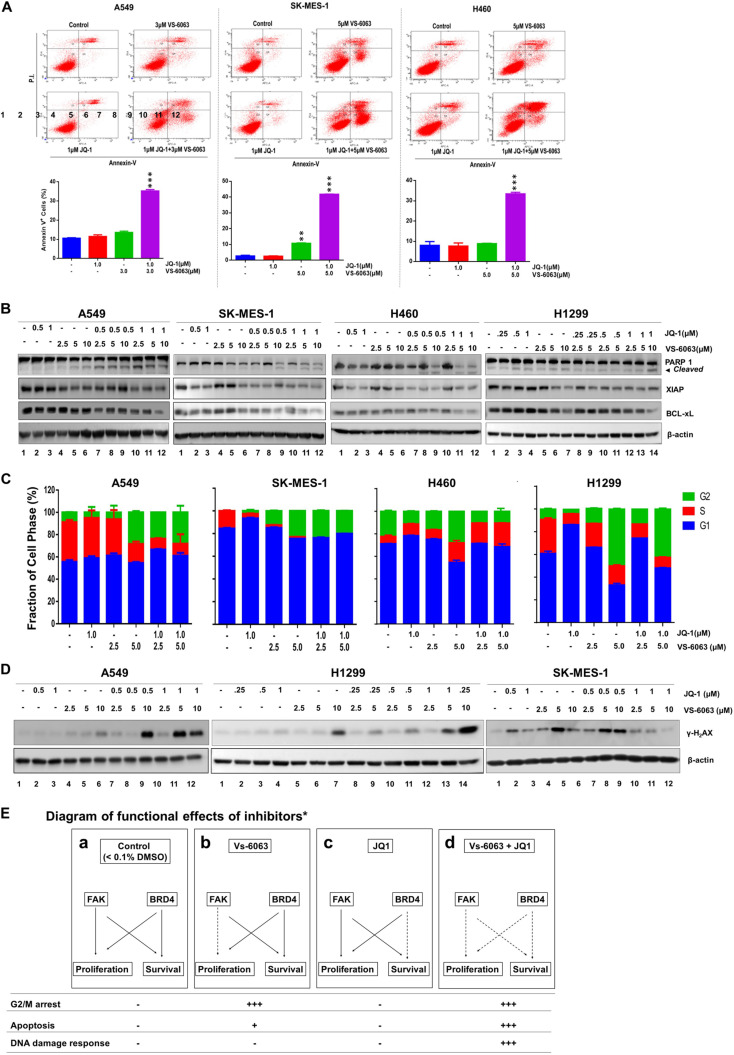
The functional effect of co-disruption of FAK and BRD4 in NSCLC cells. **(A)** Analysis of inhibitor effect on cell survival. A549, SK-MES-1 and H460 cells were treated with indicated doses or combination of inhibitors for 48h, followed by estimates of the percentage of cells stained with propidium iodide and Annexin V on flow cytometry. Top panel, histogram plot of cell staining populations. Bottom panel, estimate of percentage of Annexin V^+^ cells. **(B)** Biochemical analyses of inhibitor effect on cell survival/death pathway. Tumor cells were treated with varying doses of JQ1 and VS-6063 for 24 h, and immunoblotted for PARP1, XIAP, and Bcl-xL. **(C)** Effect on cell cycle. Tumor cells were starved and treated with inhibitors for 48 h before the flow cytometry analysis with propidium iodide. **(D)** Effect on DNA damage. Tumor cells were treated with inhibitors for 24 h, and blotted for phosphorylated histone H2Ax. **(E)** Diagram of differential functional effects of FAK and BRD4 inhibitors. β-actin was blotted for equal protein loading **(B**,**D)**. **(A,C)** Values: Mean ± SEM (*n* = 3) *p < 0.05; **p < 0.01; and ***p < 0.005.

We subsequently examined the biological basis for the collaborative effect of VS-6063 and JQ1 on the viability of NSCLC cells. As shown in Figure 3A, based on our flow cytometry-based detection of fractions of Annexin V^+^ cells, VS-6063, and JQ1 at sub-optimum doses synergistically induced apoptotic cell death in all three cell lines (A549, SK-MES-1, H460, and H1299), and marked changes in levels of cleaved PARP1 and two key pro-survival mediators, XIAP and Bcl-xl (Figure 3B). In contrast, there was an effect of VS-6063, but not JQ1, on tumor cell transition through the G_2_/M phase in three NSCLC cell lines (A549, SK-MES-1, and H1299) (Figure 3C). However, there was a strong cooperative effect of VS-6063 and JQ1 on DNA damage response, as indicated by increased S^139^ phosphorylation of histone H2AX (Figure 3D). Collectively, these data indicate that a simultaneous inhibition of the integrin/FAK axis and BRD4 is highly effective in inducing tumor cell death and DNA damage response, but not proliferation (Figure 3E).

### Signaling Effect of Co-inhibition of the Integrin-FAK Axis and BRD4

**FIGURE 4:**
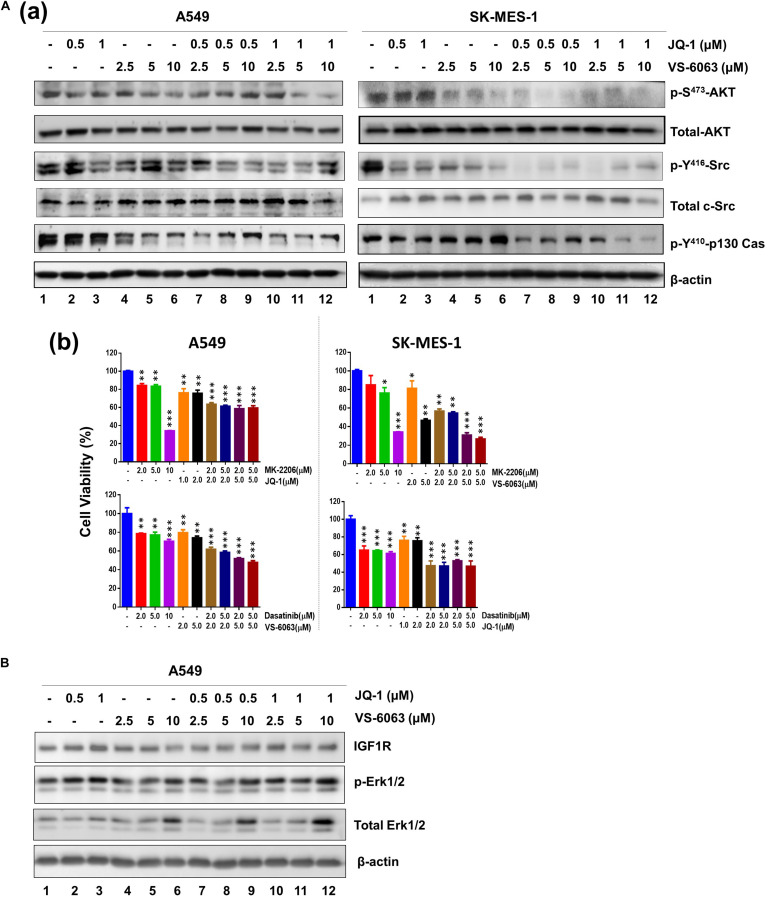
The signaling effect of co-inhibition of the integrin/FAK and BRd4/c-Myc axes in NSCLC cells. A549 and SK-MES-1 cells were treated with chemical inhibitors for 24 h, and analyzed for panel **A(a)** phosphorylated and total of Akt and c-Src, Erk1/2 and p130Cas; **(b)** Combined effect of FAK, BRD4, Akt and c-Src inhibitors in tumor cells. **(B)** Effect on activation of IGFR and Erk1/2 pathway. Tumor cells were treated as above, and blotted for indicated protein kinases. *p < 0.05; **p < 0.01 and ***p < 0.005.

We next investigated the molecular basis for functional cooperation between the integrin-FAK axis and BRD4 in NSCLC cells. Our data showed there was a notable cooperative effect of FAK and BRD4 inhibitors on the activation of Akt- and c-Src/p130 complex-mediated signaling pathways, as detected by phosphorylation of S473, p-Y416, and Y410 residues, respectively (Figure 4A). In contrast, this inhibitor combination appeared to have minimal effect on the MAPK activation (Figure 4B). These data demonstrate that the VS-6063/JQ1 combination impairs tumor cell survival largely by disrupting Akt- and c-Src/p130 complex-mediated signaling.

### A Link to the EMT Program, Cell Spreading and Stemness

**FIGURE 5:**
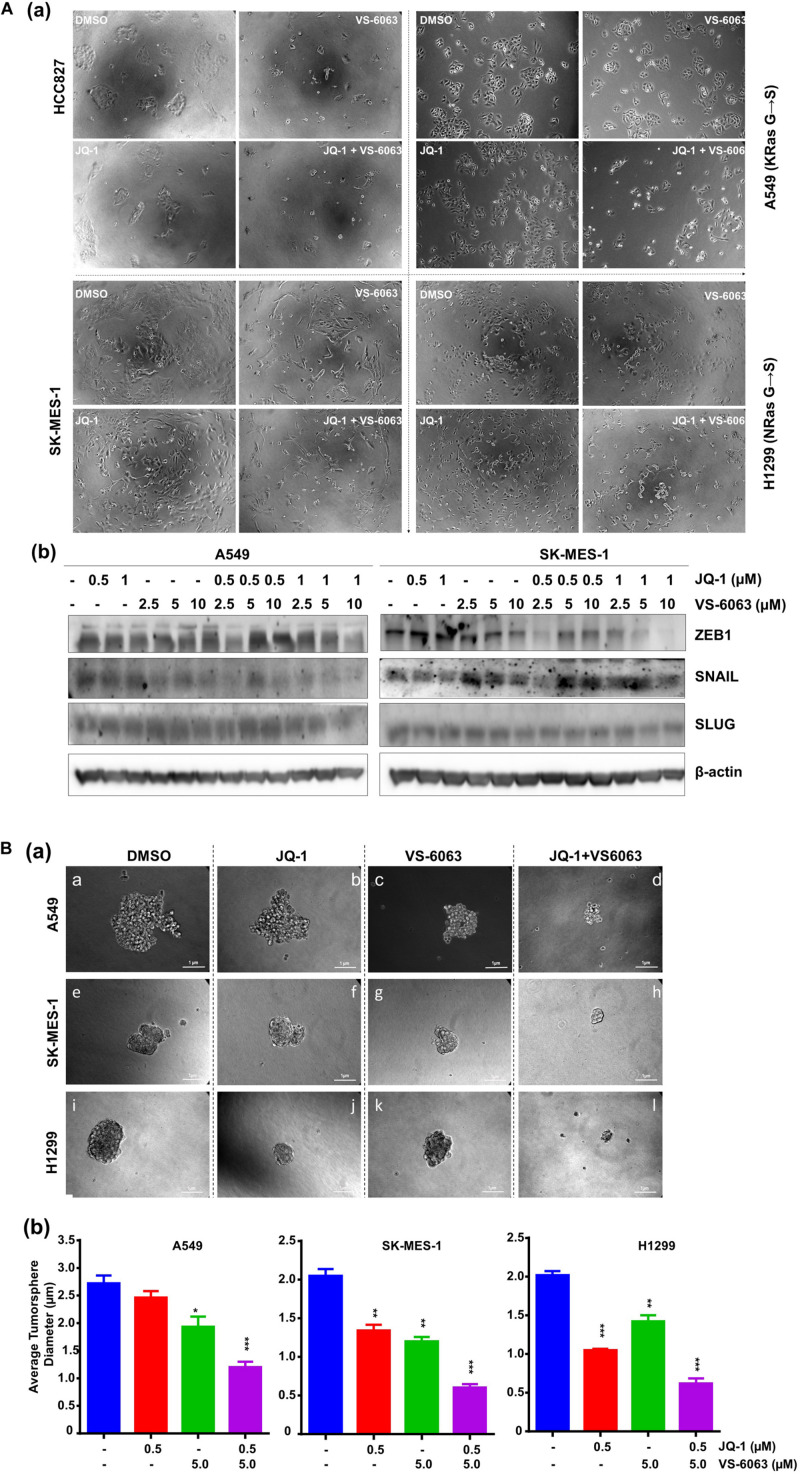
The inhibitor effect on tumor cell morphologies and stemness. **(A)** Tumor cells were seeded in 24 or 6 well plates, treated with treated with 0.5 μM JQ1 and/or 3.0 μM VS-6063 for 24 h and imaged **(a)** or indicated doses of inhibitors, lysed in RIPA buffer, and blotted with indicated antibodies **(b)** β-actin was blotted for equal protein loading. **(B)** Effect on tumorsphere formation. 1.0 × 10^3^ tumor cells were seeded into single well of 24-well ultra-low adhesion plate overnight, followed by treatment with 0.5 and 3.0 μM VS-6063 for 5 days in triplicates, and imaged microscopely. **(a)** Representative images of tumorspheres; **(b)** Differences in average diameters of tumorspheres between treatment groups. Values: Mean ± SEM (*n* = 3). *p < 0.05; **p < 0.01; and ***p < 0.005.

Given the strong link between integrins and maintenance of epithelial cell-like morphologies in NSCLC (Sulzmaier et al., 2014), we examined the impact of co-inhibition of the integrin/FAK and BRD4/c-Myc axes on cell adhesion and EMT. As shown in Figure 5Aa, JQ1 induced EMT-like morphological changes in multiple NSCLC cell lines, reminiscent of the EMT induction. In line with this effect, there was a marked decrease in level of two major EMT-inducing transcription factors, Snail and ZEB1 (Figure 5Bb). However, there was also decreased cell spreading, indicating a concomitant suppression of integrin functions. Moreover, co-inhibition of FAK and BRD4/c-Myc markedly decreased the size of tumorspheres formed under stem cell culture condition (Figure 5B). Collectively, these data demonstrate that the co-target coincides with disruption of the EMT-like program, cell-ECM adhesion and a decrease in capacity of NSCLC cancer stem cells.

### Clinical Relevance of the Co-deregulation of the Integrin-FAK Axis and the BRD4-c-Myc Axis

**FIGURE 6:**
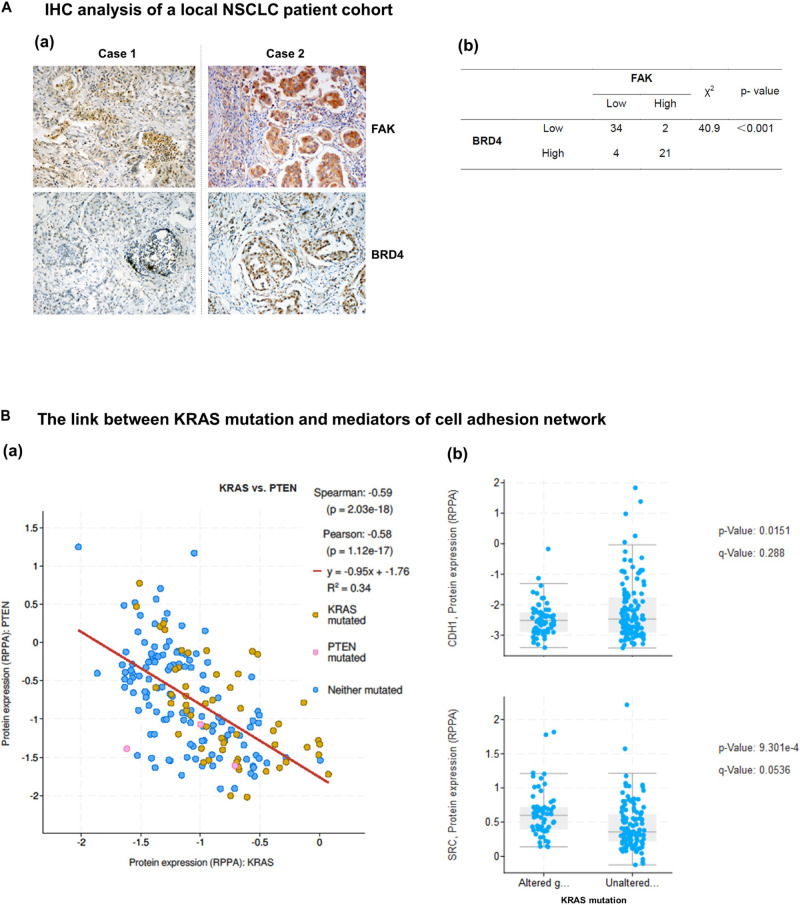
Co-expression and activation of integrin-FAK and BRD4/c-Myc axes in NSCLC patient cohorts. Patient biopsies and *in vivo* evaluation of the co-inhibition of FAK and BRD4. **(A)** Analysis of co-upregulation of FAK and BRD4 in adenocarcinoma tissues from a local NSCLC patient cohort. **(B)** Differential expression of integrin-linked PTEN, E-cadherin and c-Src in patient biopsies with respect to KRAS status in the TCGA cohort. *p* values were indicated. Scale bar: 1 μm. **(a)** Representative images of antibody staining of FAK and BRD4 in patient biopsies with or without co-upregulation of two proteins (Cases 1 and 2). **(b)** The X square analysis of degree of co-upregulation of FAK and BRD4 in the patient cohort.

We next examined the extent of co-regulation of the integrin-FAK and the BRD4-c-Myc axes by performing IHC analyses of human NSCLC patient biopsies. As shown in Figure 6A and Supplementary Table S1, BRD4 and FAK exhibit an upregulation in 34% (21 out of 61) lung adenocarcinomas. In the TCGA cohort, expression of PTEN, a negative regulator of the integrin-FAK signaling, appeared downregulated or mutated in a large portion of KRAS mutated NSCLC patient biopsies, compared to their counterpart (Figure 6B). In addition, E-cadherin, not c-Src, exhibited a decreased expression in KRAS mutated patient biopsies. Together, these data suggest the integrin/FAK signaling, along with EMT program, is activated in KRAS mutated NSCLC tumors.

**FIGURE 7:**
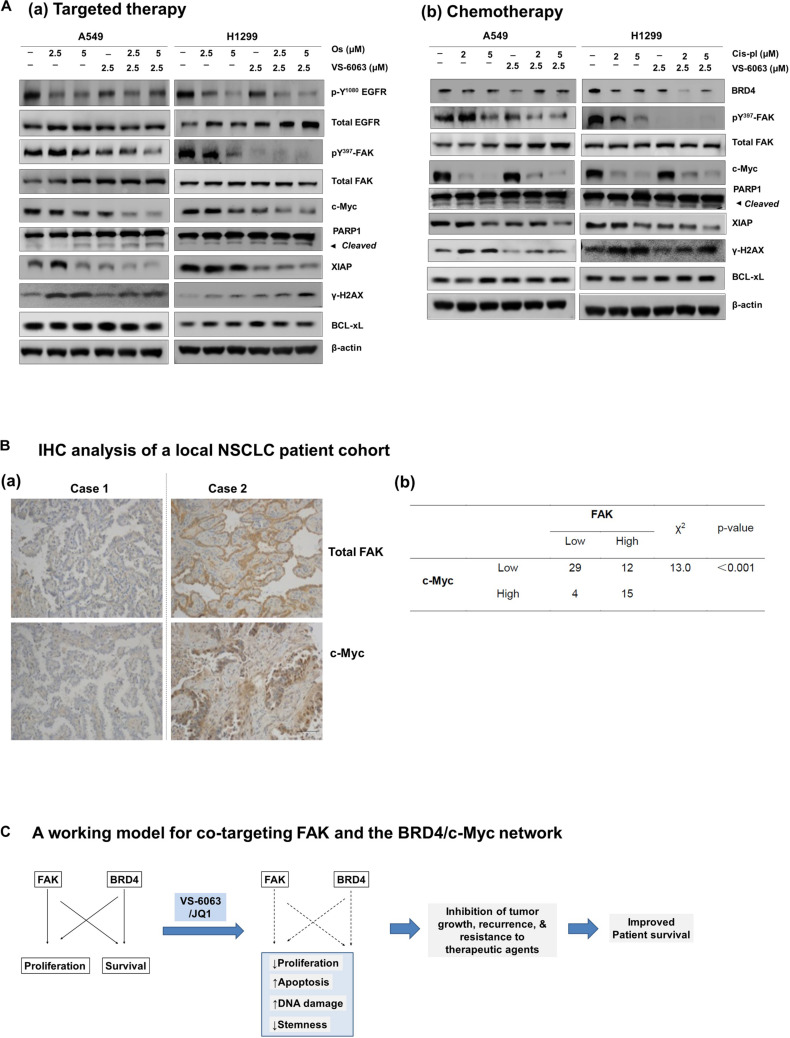
Comparison of effects of the FAK/BRD4 inhibitors and therapeutic agents in NSCLC cells. **(A)** Analysis of effect of varying doses of FAK and Osimertinib **(a)** or Cisplatin **(b)** on activation of EGFR, FAK, c-Myc and apoptotic cell death and DNA damage response. **(B)** Analysis of co-upregulation of FAK and c-Myc in adenocarcinoma tissues from a local NSCLC patient cohort. **(C)** The working model for co-targeting FAK and BRD4 in NSCLC.

Finally, we compared the effect of VS-6063/JQ1 combination with currently used therapies in terms of tumor cell viability. As shown in Supplementary Figure S4, the impact of the VS-6063/JQ1 combination on cell viability appeared equal to that of VS-6063 with Cisplatin, an anti-NSCLC chemotherapeutic agent for treating lung cancer or Osimertinib, a third generation of EGFR inhibitor. In addition, there was a corresponding decrease in c-Myc level, aside from expected change in pro-apoptotic XIAP, PARP1 cleavage and DNA damage response (Figure 7A). Moreover, c-Myc protein exhibited a co-upregulated with FAK in 25% (15 out of 61) lung adenocarcinomas from our local cohort (Figure 7B). Collectively, these data reveal a strong similarity between the concurrent inhibition of the integrin-FAK and BRD4-c-Myc axes and current therapies in NSCLC in terms of their anti-tumor cell growth effects.

## Discussion

Here we describe a close crosstalk between the integrin/FAK-dependent pathway and the BRD4-associated epigenetic network in human NSCLC in the context of KRAS mutations. Our analysis of the TCGA database indicates a strong upregulation of multiple integrins in a mutually exclusive manner, a change that is strongly linked to poor clinical prognosis in the TCGA lung adenocarcinoma cohort. Our analysis with chemical inhibitors and gene knockdown also reveals that the integrin-FAK axis and epigenetic network (BRD4 and HDAC) cooperatively promote tumor cell survival, but not cell cycle progression. These functional impacts, to a large extent, are attributable to the regulation of p130Cas/c-Src complex- and the Akt/XIAP/Bcl-xl axis-mediated signal transduction. In addition, suppression of tumor cell viability by the co-inhibition of FAK and BRD4 is nearly equivalent to the targeted or chemo- therapeutic agents being employed for treatment of NSCLC. Overall, our data support the therapeutic potential of co-targeting the integrin/FAK-dependent pathway and the BRD4-associated epigenetic network for KRAS mutated NSCLC (Figure 7C).

### Novel Insights Into Integrin/FAK-Dependent Signaling in NSCLC

Data from our current study consistently support a critical role of multiple integrins in development of NSCLC malignancy. To date, the functional link between the integrin-dependent signaling and the NSCLC malignancy largely comes from the αvβ6 integrin dependence of KRAS mutated adenocarcinomas (Agochiya et al., 1999; Rodriguez-Pinilla et al., 2007). Our analysis of TCGA cohort indicates that FAK, the key effector of integrin signaling, appears largely deregulated at the signaling level, in contrast to gene amplification in other epithelia-origin cancers such as ovarian and colon cancers (Xu et al., 2017). In line with this evidence is the inverse association between KRAS and PTEN in lung adenocarcinoma (Figure 5). The nature of PTEN as a potential phosphatase of FAK offers another layer of importance for FAK activation in KRAS mutated NSCLC. The EGFR mutation also appears to drive NSCLC addiction to the integrin-FAK axis, based on our inhibitor analysis with HCC827 (Figure 1C). Meanwhile, there is still a possibility that FAK may act downstream of the integrin-RhoA pathway during tumor progression or metastasis of NSCLC. Once our *in vitro* and clinical observations are validated *in vivo*, they should serve as additional support for the pro-tumorigenic role of the integrin-FAK signaling axis in human NSCLC.

### The Link Between the Integrin/FAK Pathway and BRD4-Associated Epigenetic Network

Our current study strongly supports a collaborative role of the integrin/FAK signaling axis and BRD4-associated epigenetic network in NSCLC tumor cells. As an epigenetic regulator and transcription factor, BRD4, along with its chemical inhibitor JQ1, is strongly implicated in regulation of cell proliferation, survival and tumor growth in KRAS mutated NSCLC in a c-Myc-dependent manner (Shimamura et al., 2013; Bouillez et al., 2016). Our data indicates that this effect is apparent in two squamous carcinoma-like NSCLC cell lines, SK-MES-1 and H460, but not A549 cells (Figure 1). However, JQ1 appears to have a minimal effect in A549 cells, where EGFR amplification and KRAS mutation occur (Figures 3–7). In contrast, we found that the viability of is synergistically disrupted by co-targeting of the integrin/FAK signaling axis and BRD4-associated epigenetic network and a similar trend is detected across a large panel of NSCLC cell lines (Figure 1). This broad efficacy is also supported by the co-upregulation of FAK and c-Myc in nearly a quarter of the NSCLC adenocarcinomas and at mRNA level in our NSCLC patient cohort (Figures 6, 7). Moreover, the collaboration of these distinct axes in regulation of cell survival via XIAP and Bcl-xL in NSCLC is consistent with our recent study of another epithelia-origin cancer (Xu et al., 2017), further supporting the notion that co-targeting of FAK and c-MYC represents a line of synthetic lethal inhibition in human epithelia-origin cancers, regardless of genomic status of integrin-FAK axis and c-Myc and context of oncogenic activation.

Besides the impacts on cell survival and tumor growth, inhibition of the integrin-FAK axis and BRD4-linked epigenetic network may alter cytokine pathways and tumor microenvironments. In line with this notion, both of these networks are implicated in inflammatory process during tumor development and progression in various cancer types, including NSCLC (Belkina et al., 2013; Jiang et al., 2016; Adeegbe et al., 2017). In this regard, the co-inhibition of FAK and BRD4 may lead to impaired activation of dynamics of T regulatory cell populations and IL8-linked NF-KB network in NSCLC adenocarcinomas, including those with KRAS mutations (Sunaga et al., 2012; Adeegbe et al., 2017). This possibility remains to be addressed in the future study with the PDX model for human NSCLC.

One potential complexity between the integrin-FAK axis and BRD4 is a direct role of BRD4 in regulation of integrin expression and function. This notion is supported our detection of the effect of JQ1 on ECM-integrin-mediated cell spreading in NSCLC cells, beside EMT (Figure 5A). This observation is in line with the evidence that small GTPase Rac1, one of the key downstream effectors of integrin signaling, is associated with maintenance of BRD4 protein stability (Zhang et al., 2017). In addition, BRD4 is implicated in promoting expression of β1 integrin at the transcriptional level (Kim et al., 2007). Hence, part of the BRD4-associated effect on NSCLC may stem from its regulation of the integrin-dependent signaling. Overall, BRD4 impacts tumor growth in NSCLC in both Myc-dependent and independent manners (Takahara et al., 2017).

### Therapeutic Potential of Co-targeting the Integrin-FAK Axis and BRD4-Linked Network

Our study also reveals that the effect of co-inhibition of integrin/FAK pathway and BRD4 in NSCLC is not only equivalent to that of VS-6063 in combination with cisplatin or Osimertinib. The efficacy of this co-inhibition appears independent of KRAS or EGFR amplification status (Figures 1, 7). Compared to most of current therapies, this potential combinatorial therapy appears to have broader efficacy, ranging from tumors with deregulation of receptor tyrosine kinases (RTKs) to those with the RAS mutation, as the combined effect of VS-6063 and JQ1 converges at c-Myc (Delmore et al., 2011; Kandela et al., 2015), thereby bypassing the need for a direct inhibition of KRAS-mediated pathways (e.g., KRAS, MEK or Erk1/2). As a result, co-targeting FAK and BRD4 may serve as an alternative therapy to current targeted therapy for EGFR mutation such as Gefitinib, Erlotinib, and Osimertinib, as well as potential inhibitors of KRAS with carrying G → C mutation in NSCLC (Nadal et al., 2015).

While our study focuses on the effect on the adenocarcinoma subtype of NSCLC, the co-inhibition of FAK and BRD4 may be effective for the squamous subtype of NSCLC as well. This notion is supported by the strong role of the integrin-dependent signing in tumor cell survival and growth in this subtype (Marinkovich, 2007; Prudkin et al., 2009). This co-targeting may also abrogate recurrence of the disease, as it synergistically impairs the ability of cancer stem cells to form tumorspheres (Figure 5). Additionally, it is worth noting that we also conducted preliminary *in vivo* analysis of the anti-tumor efficacy for the VS-6063 and JQ1 combination in xenograft model. Our initial treatment with sub-optimal does of JQ1 and VS-6063 detected moderate inhibitory effect on tumor growth in the lung of immune-compromised nude mice over a 1-week span (data not shown). However, due to appearance of diarrhea, we subsequently reduced the dose by 15% but failed to detect a marked effect (data not shown). Nonetheless, the *in vivo* efficacy of this inhibitor combination still needs to be evaluated with preclinical models in the future.

In summary, our study has revealed that the integrin/FAK signaling axis and BRD4-associated epigenetic network act cooperatively to support NSCLC cell survival and ability to form tumorspheres. To a large extent, this effect is linked to a cooperative impact on Src/p130Cas and Akt/XIAP/Bcl-xl-dependent signaling, EMT and integrin functions in tumor cells. The chemical inhibitor-based co-targeting may lead to inhibition of tumor cell growth in both Myc-dependent and independent manners, while having a minimal impact on RAS/MEK/ERK-dependent signaling. Hence, our study supports co-targeting of the integrin-FAK pathway and BRD4 as a promising line of synthetic lethal-type therapy for NSCLC, regardless of the state of EGFR or KRAS.

## Data Availability Statement

All datasets presented in this study are included in the article/Supplementary Material.

## Author Contributions

YZ, KC, BX, JS, JQ, SS, YY, HL, TJ, RG, and YW participated in the data collection and analysis. ZL, XW, J-AH, and XY participated in the design of the study. YZ, KC, BX, JS, JQ, and XY participated in the writing of the manuscript and the interpretation of the data. All authors read and approved the final manuscript.

## Conflict of Interest

The authors declare that the research was conducted in the absence of any commercial or financial relationships that could be construed as a potential conflict of interest. The handling editor declared a shared affiliation with one of the authors XY at the time of review.

## Supplementary Material

FIGURE S1 Association between deregulated expression of integrins and Co-activation of EGFR and KRAS in NSCLC. **(A)** The oncoprint map of expression of major integrins and status of KRAS and EGFR in adenocarcinomas in the TCGA NSCLC cohort (pan alters, Nature 2014). **(B)** Association between co-activation of EGFR and KRAS and clinical outcomes of patients in the TCGA cohort. **(a,c,e)**: Kaplan-Meier’s curve for overall patient survival. **(b,d,f)**: Kaplan-Meier’s curve for disease/progression-free patient survival. **(a,b)**: KRAS activation; **(c,d)**: EGFR deregulation; Co-deregulation of KRAS and EGFR. and clinical association. Logrank Test *p* values were indicated.

FIGURE S2 Chemical inhibitor-based screening for collaborators of integrin-FAK-dependent pathways. A549 cells were seeded in 96-well plates and treated with indicated does of inhibitors for 73 h, followed by MTT assay. The combined effect of VS-6063 with five different classes of chemical inhibitors were shown. **(A)** Inhibitors of receptor tyrosine kinases (RTKs). **(B)** Inhibitors of PI3K/Akt pathway. **(C)** Inhibitors of cancer stem cell-associated pathway. **(D)** Inhibitors of epigenetic network. **(E)** Inhibitors of Wnt pathway.

FIGURE S3 Effect of additional FAK and BRD4 inhibitors on viability of NSCLC cells. Tumor cells were treated with additional inhibitors of FAK and BRD4, including VS-4716 and IBET-762, followed by analysis of effect on cell viability with MTT assay. Cell viability: calculated as percentage of viable cells relative to 0.1% DMSO control, Mean ± SEM (*n* = 3). ^∗^*p* < 0.05; ^∗∗^*p* < 0.01; and ^∗∗∗^*p* < 0.005.

FIGURE S4 The link between the FAK/BRD4 co-inhibition and clinically used therapeutic agents. A549, SK-Mes-1, and H1299 cell lines were treated with varying doses of indicated inhibitors for 72 h, followed by analysis of cell viability via MTT assay. Cell viability: calculated as percentage of viable cells relative to 0.1% DMSO control, Mean ± SEM (*n* = 3).

TABLE S1 Demographic characteristics of the NSCLC patient cohort. The cohort was subjected to evaluation for co-expression of FAK, BRD4 and c-Myc in primary tumors by IHC analysis described in Figures 6, 7.
