# Supplementary figures and images for: Epigenetic Input Dictates the Threshold of Targeting of the Integrin-Dependent Pathway in Non-small Cell Lung Cancer
Source: Front Cell Dev Biol. 2020 Jul 22;8:652. doi: 10.3389/fcell.2020.00652 (PMC7387701; doi:10.3389/fcell.2020.00652)

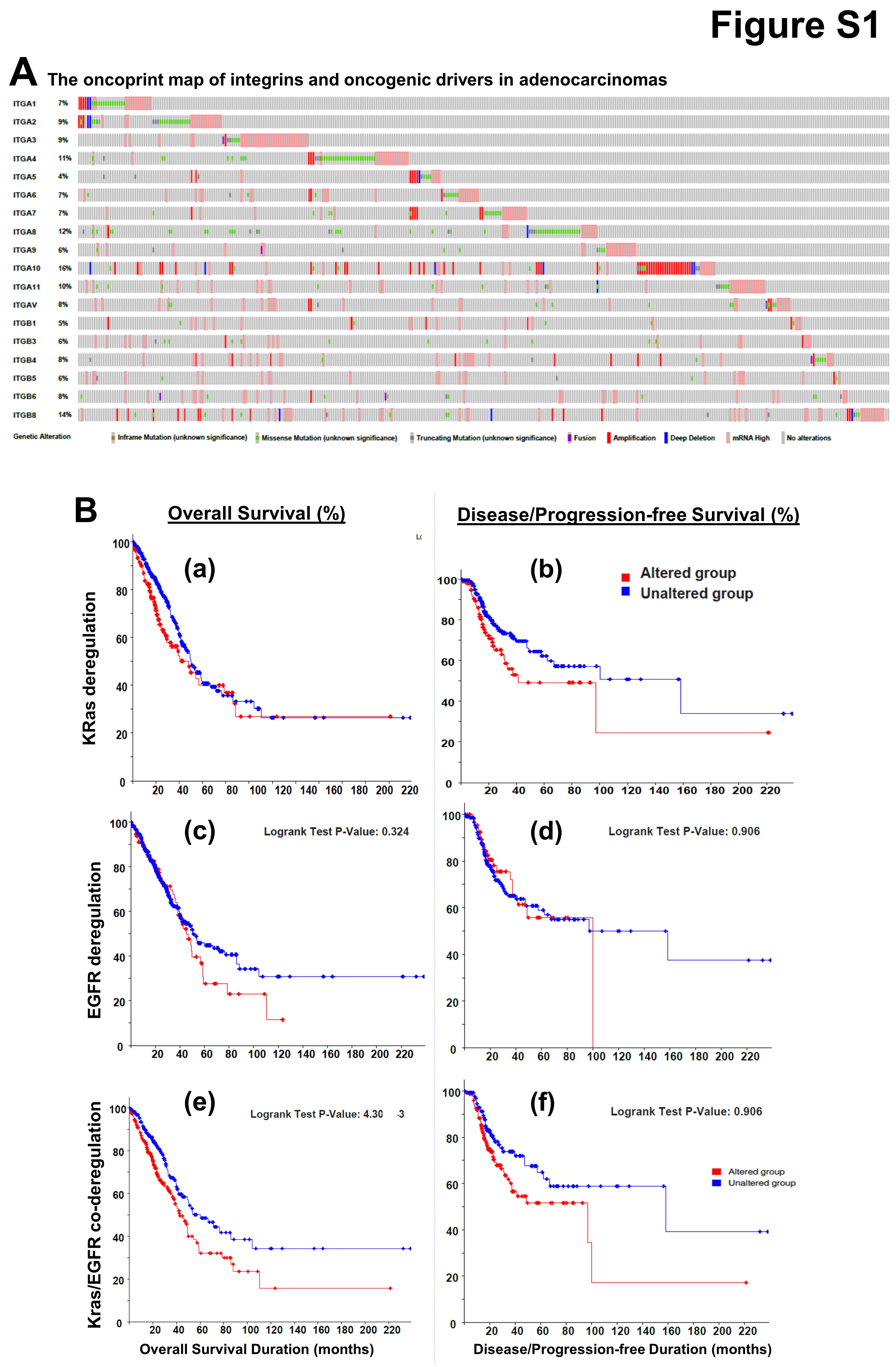

Supplement: FIGURE S1 — Association between deregulated expression of integrins and Co-activation of EGFR and KRAS in NSCLC. (A) The oncoprint map of expression of major integrins and status of KRAS and EGFR in adenocarcinomas in the TCGA NSCLC cohort (pan alters, Nature 2014). (B) Association between co-activation of EGFR and KRAS and clinical outcomes of patients in the TCGA cohort. (a,c,e): Kaplan-Meier’s curve for overall patient survival. (b,d,f): Kaplan-Meier’s curve for disease/progression-free patient survival. (a,b): KRAS activation; (c,d): EGFR deregulation; Co-deregulation of KRAS and EGFR. and clinical association. Logrank Test p values were indicated. [file Image_1.jpg]

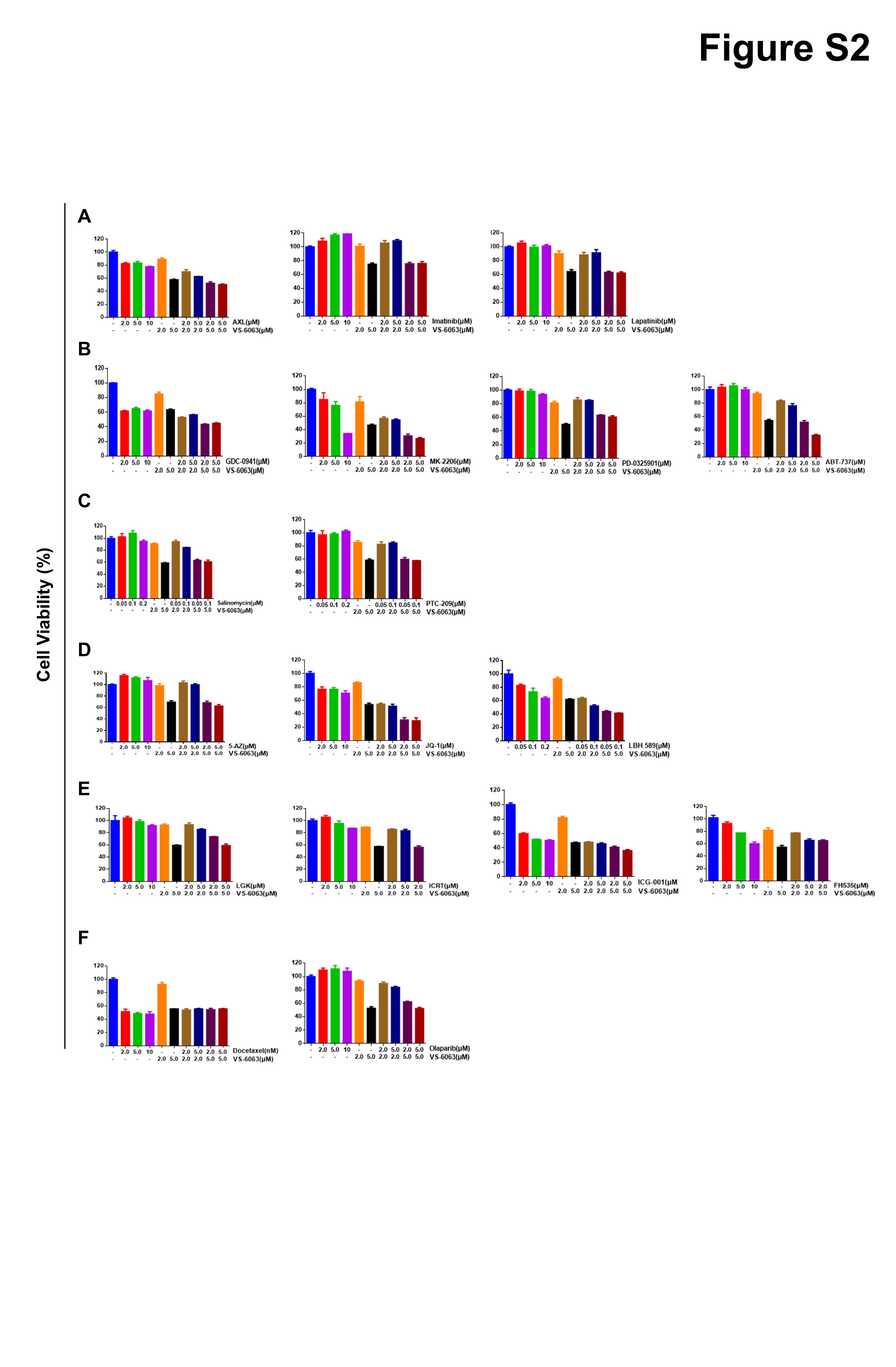

Supplement: FIGURE S2 — Chemical inhibitor-based screening for collaborators of integrin-FAK-dependent pathways. A549 cells were seeded in 96-well plates and treated with indicated does of inhibitors for 73 h, followed by MTT assay. The combined effect of VS-6063 with five different classes of chemical inhibitors were shown. (A) Inhibitors of receptor tyrosine kinases (RTKs). (B) Inhibitors of PI3K/Akt pathway. (C) Inhibitors of cancer stem cell-associated pathway. (D) Inhibitors of epigenetic network. (E) Inhibitors of Wnt pathway. [file Image_2.jpg]

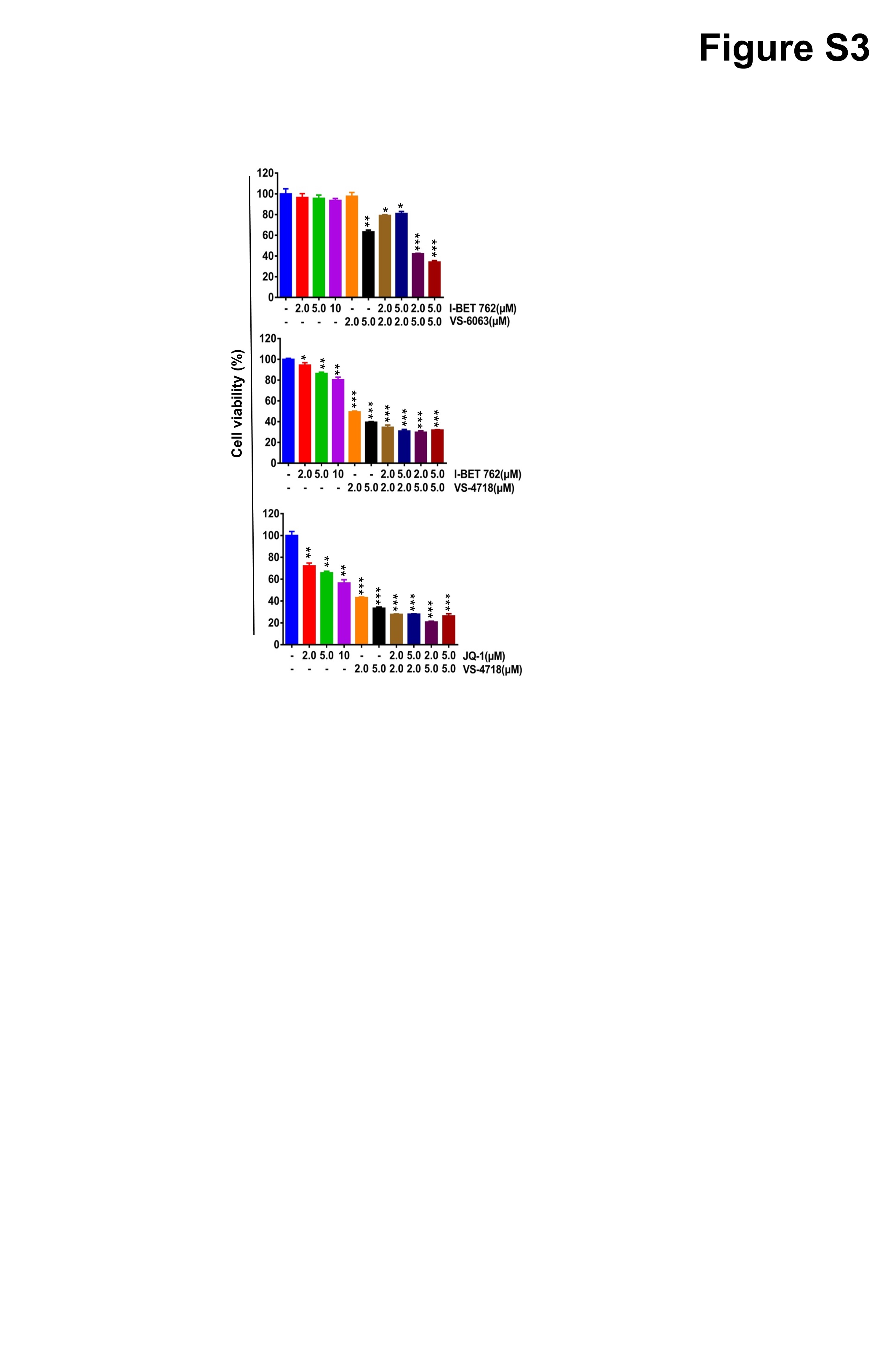

Supplement: FIGURE S3 — Effect of additional FAK and BRD4 inhibitors on viability of NSCLC cells. Tumor cells were treated with additional inhibitors of FAK and BRD4, including VS-4716 and IBET-762, followed by analysis of effect on cell viability with MTT assay. Cell viability: calculated as percentage of viable cells relative to 0.1% DMSO control, Mean ± SEM (n = 3). ∗p < 0.05; ∗∗p < 0.01; and ∗∗∗p < 0.005. [file Image_3.jpg]

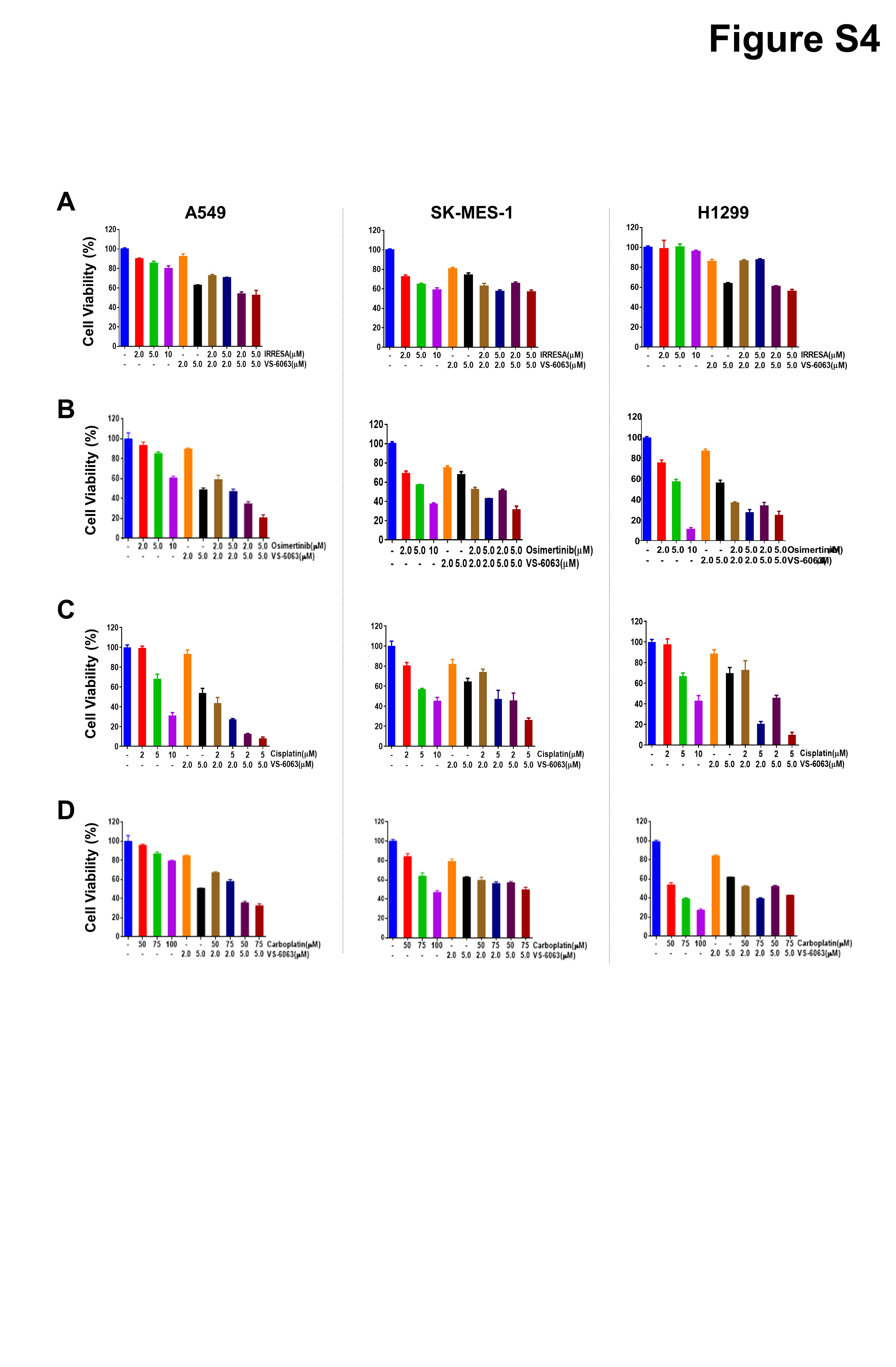

Supplement: FIGURE S4 — The link between the FAK/BRD4 co-inhibition and clinically used therapeutic agents. A549, SK-Mes-1, and H1299 cell lines were treated with varying doses of indicated inhibitors for 72 h, followed by analysis of cell viability via MTT assay. Cell viability: calculated as percentage of viable cells relative to 0.1% DMSO control, Mean ± SEM (n = 3). [file Image_4.jpg]
