# Supplementary material for: Epigenetic Input Dictates the Threshold of Targeting of the Integrin-Dependent Pathway in Non-small Cell Lung Cancer
Source: Front Cell Dev Biol. 2020 Jul 22;8:652. doi: 10.3389/fcell.2020.00652 (PMC7387701; doi:10.3389/fcell.2020.00652)
Supplement: TABLE S1 — Demographic characteristics of the NSCLC patient cohort. The cohort was subjected to evaluation for co-expression of FAK, BRD4 and c-Myc in primary tumors by IHC analysis described in Figures 6, 7. [file Table_1.pdf]

# Table S1

## Clinical characteristics of patients with upregulation of FAF or c-Myc proteins

| Characteristics | FAK (N) | C-Myc (N) |
|---|---|---|
| Gender | | |
| Male | 11(40.7%) | 7(36.8%) |
| Female | 16(59.3%) | 12(63.2%) |
| P value | 0.895 | 0.606 |
| Age(years) | | |
| <60 | 15(55.6%) | 9(47.4%) |
| ≥60 | 12(44.4%) | 10(52.6%) |
| P value | 0.311 | 0.65 |
| Maximum tumor diameter(cm) | | |
| <3 | 9(33.3%) | 15(78.9%) |
| ≥3 | 18(66.7%) | 4(21.1%) |
| P value | 0.34 | 0.041 |
| Histological differentiation | | |
| High or middle | 20(74.1%) | 14(73.7%) |
| Low | 7(25.9%) | 5(26.3%) |
| P value | 0.299 | 0.405 |
| Necrosis | | |
| No | 23(85.2%) | 15(78.9%) |
| Yes | 4(14.8%) | 4(21.1%) |
| P value | 0.261 | 0.052 |
| Lymphatic metastasis | | |
| No | 15(55.6%) | 8(42.1%) |
| Yes | 12(44.4%) | 11(57.9%) |
| P value | 0.379 | 0.034 |
| Ki-67 | | |
| <30 | 6(22.2%) | 4(21.1%) |
| ≥30 | 21(77.8%) | 15(78.9%) |
| P value | 0.036 | 0.088 |
